# Lubiprostone plus polyethylene glycol electrolyte lavage solution (PEG-ELS) versus PEG-ELS for bowel preparation in chronic constipation: a randomized controlled trial

**DOI:** 10.1038/s41598-023-43598-6

**Published:** 2023-09-27

**Authors:** K. Tangvoraphonkchai, W. Manasirisuk, K. Sawadpanich, T. Suttichaimongkol, P. Mairiang

**Affiliations:** 1 Gastroenterology and Hepatology Unit, Department of Medicine, Faculty of Medicine, Khon Kaen University, Khon Kaen, Thailand; 2 GI Endoscopy Srinagarind Center of Excellence, Srinagarind Hospital, Khon Kaen, Thailand; 3 Srinagarind Hospital, KKU, Khon Kaen, Thailand

**Keywords:** Colonoscopy, Colorectal cancer

## Abstract

Colonoscopy is considered the standard procedure for early detection and prevention of colorectal cancer. Adequate bowel cleansing is an important determinant of the efficacy of colonoscopy screening. Currently, there is no standard method of bowel preparation for patients with chronic constipation. The aim was to access the rate of adequate bowel cleansing achieved using split-dose polyethylene glycol electrolyte lavage solution (PEG-ELS) plus lubiprostone in comparison with split-dose PEG-ELS alone. A single-centre, endoscopist-blinded, randomized controlled trial was conducted. Seventy-eight constipated patients aged 18–75 years who were indicated for colonoscopy in the gastroenterology unit of Srinagarind Hospital, Khon Kaen University, between February 2020 and February 2021 were randomly allocated to receive either split-dose PEG-ELS in combination with lubiprostone (N = 39) or split-dose PEG-ELS alone (N = 39) before colonoscopy. Adequate bowel cleansing was defined as an Ottawa bowel preparation score ≤ 7. The rate of adequate bowel cleansing was comparable between the PEG-ELS plus lubiprostone group and the PEG-ELS alone group (50% vs. 52.9%, *p* value = 0.81) with a relative risk of 1.13 (95% CI = 0.43–2.91). There were no significant differences in adenoma detection rate (41.2% vs. 35.3%, *p* value = 0.62), adverse events, acceptance, compliance, or patient satisfaction between the 2 groups. No additional benefit to successful bowel preparation was achieved by the combination of lubiprostone and PEG-ELS in chronic constipation patients undergoing colonoscopy.

## Introduction

The incidence of colorectal cancer increases in Thailand annually^1^. It is most commonly diagnosed in the advanced stage; less than 50% of cases are diagnosed in stages I and II^2^. Colonoscopy is currently considered a standard and the most commonly used method for the screening, diagnosis, and treatment of disease.

High-quality colonoscopy is important for colorectal screening. The current recommendations defined a high-quality colonoscopy according to various factors, such as an adequate adenoma detection rate (ADR > 30% in men and > 20% in women) and examination-specific characteristics (complete to caecum ≥ 95%, adequate bowel preparation to detect lesions > 5 mm)^3^.

Chronic constipation affects cleansing during bowel preparation. A foreign study on the preparation of the large intestine in people with constipation showed that split-dose polyethylene glycol electrolyte lavage solution (PEG-ELS) could clean the colon by approximately 81.4%^4^; moreover, a study from Srinagarind Hospital found that using 4 L of split-dose PEG-ELS was able to clean the colon by only 73%^5^, which is less than the > 85% that defined adequate bowel preparation in the recommendations^3^.

Lubiprostone was approved by the US Food and Drug Administration in 2007. It is used to treat constipation by activating chloride channels (ClC-2 channels), which increase the amount of fluid within the intestine without severe complications^6^. The most common side effects are nausea and vomiting^7^. The recommended dose for chronic idiopathic constipation is 24 µg twice a day^6,7^.

The current guidelines do not suggest any specific bowel preparation in constipation patients^8^. Therefore, the objective of this research was to compare the proportion of adequate bowel cleansing achieved by split-dose PEG-ELS plus lubiprostone and split-dose PEG-ELS alone before colonoscopy in constipation patients.

## Patients and methods

### Study population

The study design was a single-centre prospective randomized controlled trial with approval by the Khon Kaen University Ethics Committee for Human Research based on the Declaration of Helsinki and the ICH Good Clinical Practice Guidelines (HE621471). The study protocol was registered at the Thai Clinical Trials Registry (TCTR) on 11/04/2023 (ID TCTR20230411007). Patients were scheduled for colonoscopy at the endoscopic unit of Srinagarind Hospital, Khon Kaen University, Khon Kaen, Thailand, between February 2020 and February 2021. The inclusion criteria were patients 18–75 years of age who were indicated for colonoscopy and had constipation (compatible with the ROME IV diagnostic criteria for chronic constipation or the history of Bristol stool form scale 1–2). The exclusion criteria included suspected bowel obstruction or perforation, contraindication to general anaesthesia, uncontrolled condition such as congestive heart failure, unstable angina pectoris, unstable hypertension, cardiac arrhythmia, liver failure, acute kidney injury, uncontrolled blood glucose, hypo- or hyperthyroid state, inability to stop warfarin for 3 days, inability to stop antiplatelet therapy for 7 days, history of colectomy, pregnancy or breastfeeding, history of drug allergy for PEG-ELS or lubiprostone, coagulopathy (INR > 1.5), and thrombocytopenia (< 50,000/µl).

### Outcome

The primary outcome in this study was to compare the proportion of adequate bowel cleansing by using an Ottawa bowel preparation scale (OBPS) score ≤ 7 between split-dose PEG-ELS plus lubiprostone and split-dose PEG-ELS alone before colonoscopy in constipation patients. The secondary outcomes were the adenoma detection rate, safety profiles, and patient satisfaction with lubiprostone.

### Randomization

**Figure 1:**
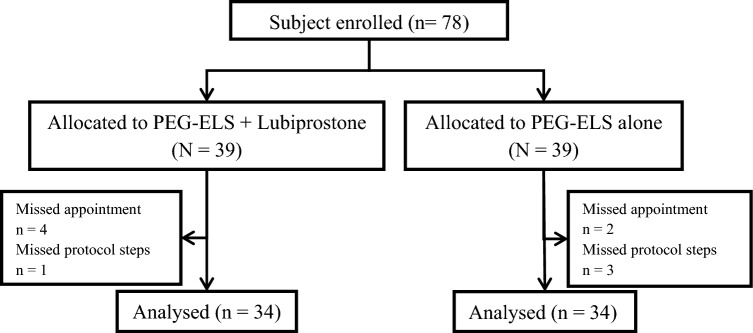
CONSORT flow diagram.

All included patients gave informed consent before study initiation. Patients were randomized to 1 of the 2 bowel cleansing regimens at an allocation ratio of 1:1. A computer-assisted randomization process with sequences was performed using the block method (block of 2 alternating with block of 4). The statistician generated the allocation sequence and assigned the participants. The health care provider enrolled the participants. All endoscopists and endoscopy nurses were blinded to participant allocation (Fig. 1).

### Bowel preparation protocol and method

The constipated patients were randomly allocated to receive either split-dose PEG-ELS in combination with lubiprostone (study group) or split-dose PEG-ELS alone (control group) before colonoscopy.

- *Study group* The study group used split-dose PEG-ELS (Niflec®) in combination with lubiprostone **(**Amitiza® 24 mcg**)** 3 times as follows: 2 days before the colonoscopy, the patient took 1 capsule of lubiprostone at 6:00 am and 1 capsule of lubiprostone at 6 pm On the day before the colonoscopy, the patient took 1 capsule of lubiprostone at 6:00 am At 6:00 pm, the patient mixed 1 sachet of PEG-ELS with 2 L of water, which had to be completely finished within 2 h. Finally, at 4:00 am on the day of the colonoscopy, the patient mixed 1 sachet of PEG-ELS with 2 L of water and completely finished it within 2 h.
- *Control group* The control group used split-dose PEG-ELS, with patients taking 1 sachet of PEG-ELS with 2 L of water at 6 pm the day before the colonoscopy and 1 sachet of PEG-ELS with 2 L of water at 4 am on the day of the colonoscopy.

Both groups were recommended to stop all laxatives before the colonoscopy for 1 week and were asked to admit IPD 1 day before the colonoscopy day. Each patient was provided with a detailed instruction card explaining the low residual diet for 3 days before colonoscopy, with only clear liquid for 1 day before colonoscopy and nothing orally after the last dose of bowel preparation.

The subjects who agreed to participate in the study underwent a blood test at an outpatient unit. On the day of the study, 10 cc blood samples were collected for complete blood count, prothrombin time, partial thromboplastin time, blood urea nitrogen, creatinine, electrolyte, calcium, magnesium, phosphate, and albumin analyses; blood pressure, height, weight, chest radiography examination and electrocardiogram were performed before receiving a laxative. Then, faeces were collected for the faecal immunochemical test (FIT). Then, another 5 cc of blood was drawn for blood tests for blood urea nitrogen, creatinine, electrolytes, calcium, magnesium, phosphate, and albumin after receiving a complete laxative (at 6.00 am). Patients received anaesthesia from an anaesthesiologist during the colonoscopy, and blood pressure and oxygen measurements were performed periodically.

### Bowel preparation score and colonoscopy methods

The endoscopists assessed bowel cleansing by the Ottawa bowel preparation score (OBPS). Adequate bowel preparation was defined as OBPS ≤ 7^9^. All colonoscopies were performed by 4 experienced endoscopists with a caecum intubation rate of more than 95%. The study results were monitored by having all 4 endoscopists assess the recorded images in each section (right, transverse/descending, sigmoid/rectum). The results of each examination were confirmed by 4 endoscopists to prevent errors. Complete colonoscopy was defined as reaching the caecum. The endoscopist recorded the caecum intubation time and withdrawal time (including duration of polypectomy time), the amount and size of the polyps, and the adverse events during colonoscopy.

### Satisfaction assessment

After the colonoscopy, the subjects completed a questionnaire to assess the satisfaction of laxative preparations using a 10-point numeric rating scale from 0 (very bad) to 10 (excellent). Acceptance was graded as none, some, or much difficulty in taking the study agent. Compliance was graded as optimal, defined as 100% intake of study agent; good, defined as ≥ 60% intake of study agent; and poor, defined as < 60% intake of study agent. When the inspectors assured that the subjects did not have any complications, they were able to return home under standardized care.

### Sample size calculation

Sample size was calculated by using two independent proportion equations. The proportion of successful bowel preparations with split-dose PEG-ELS alone was determined using data from Srinagarind Hospital, which showed that the success rate of split-dose PEG-ELS in constipated patients was 73%^5^. We assumed that the combination with lubiprostone may improve successful bowel preparation by 20%. A sample size of at least 68 subjects was required for 80% power at a 2-sided α of 0.05.

### Statistical analyses

The primary outcome in this study was compared using chi-square statistics with intention-to-treat analysis. A 95% confidence level was used, and a *p* value < 0.05 was considered statistically significant. The other continuous data in this study are expressed as the median (IQR1, IQR3). The categorical data are expressed as numbers and percentages. Statistical analyses were performed with STATA® 10.1 software.

## Results

A total of 78 patients were enrolled in this study. Ten patients were lost during this study; 6 patients missed appointments, and 4 patients missed steps in the protocol.

The demographic characteristics of the patients are shown in Table 1. Most of the patients in this study were women. Most patients in this study were not elderly or obese. The use of laxatives before colonoscopy was not different between the 2 groups, which may indicate that there was no difference in the severity of constipation between the 2 groups. Underlying diseases that were possibly associated with poor bowel preparation are also shown in Table 1**.**

**Table 1:** Demographics data.

	PEG-ELS + Lubiprostone	PEG-ELS alone
Age, median (IQR1, IQR3)	59 (61, 65)	63 (52, 69)
Female, n (%)	21 (61.8)	24 (70.6)
Body mass index (BMI), median (kg/m^2^) (IQR1, IQR3)	24.2 (21.7, 27.6)	23.2 (21.2, 25.6)
Laxative used, n (%)		
No medication	16 (47.1)	20 (58.8)
Single drug	14 (41.2)	13 (38.2)
More than 1 drug	4 (11.8)	1 (2.9)
History of cerebrovascular disease, n (%)	0	1 (2.9)
History of diabetes mellitus, n (%)	4 (11.8)	6 (17.7)
History of Parkinson’s disease, n (%)	0	1 (2.9)

**Figure 2:**
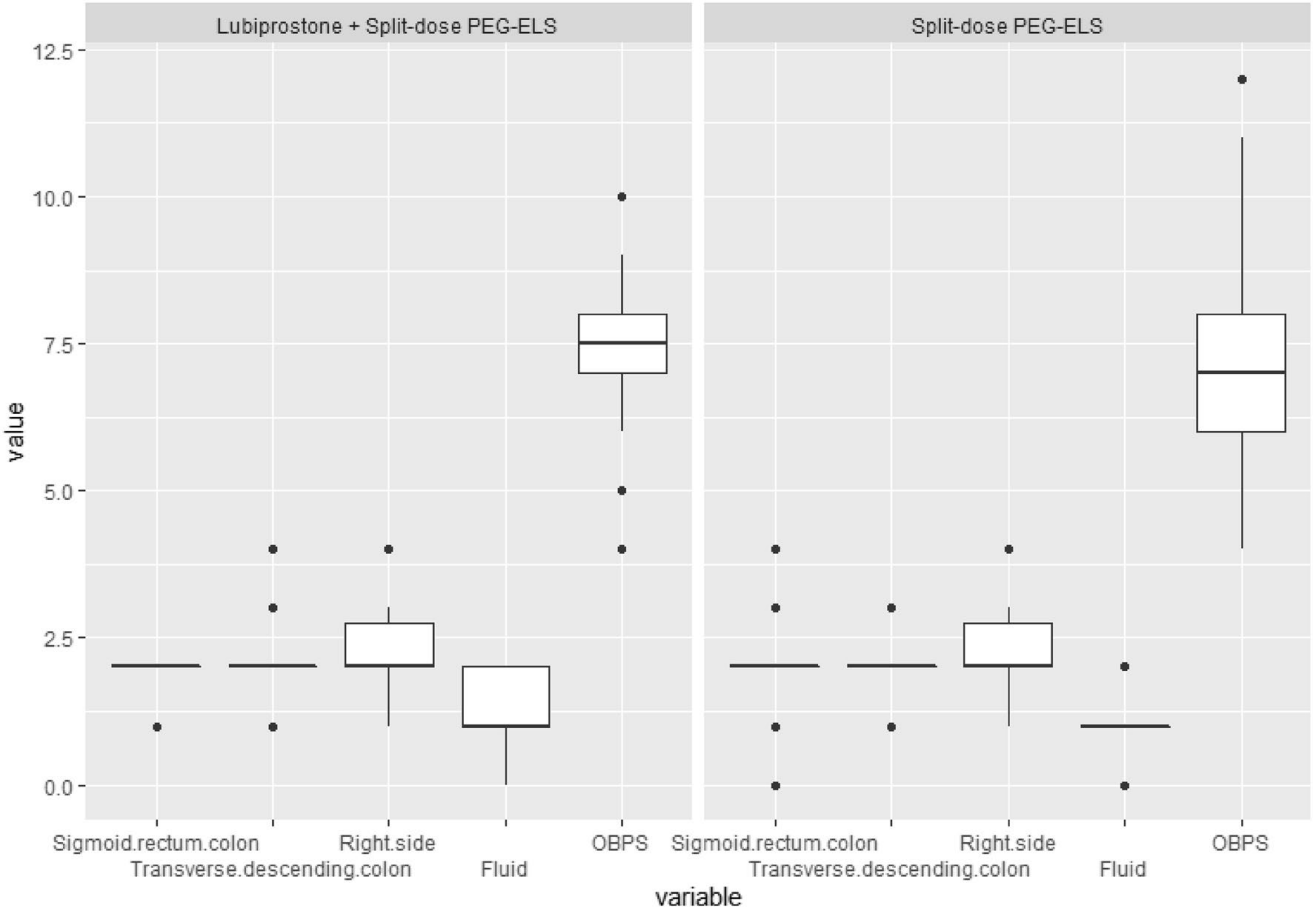
Ottawa bowel preparation scores between the 2 groups.

The quality of bowel preparation according to OBPS score is shown in Table 2**,** and the distribution of bowel cleansing segments is shown in Fig. 2. The cut-off for adequate bowel preparation was less than 8 points. There were no clinically significant differences in OBPS scores between the 2 groups (n = 17 (50%) vs. n = 18 (52.9%) *p* = 0.81), with a relative risk of 1.13 (95% CI = 0.43–2.91).

**Table 2:** Comparison of Ottawa bowel preparation scores between the 2 groups.

	PEG-ELS + Lubiprostone	PEG-ELS	*P* value
OBPS median (IQR1, IQR3)			
Sigmoid/rectum colon	2 (2,2)	2 (2,2)	0.72
Transverse/descending colon	2 (2,2)	2 (2,2)	0.84
Right colon	2 (2,3)	2 (2,3)	0.95
Fluid	1 (1,2)	1 (1,1)	0.77
** OBPS**	**7.5 (7,8)**	**7 (6,8)**	**0.84**
Adequate OBPS ≤ 7, n (%)	17 (50)	18 (52.9)	0.81

	Relative risk	95% CI	*P* value
Adequate OBPS ≤ 7	1.13	0.43–2.91	0.81

All patients underwent complete colonoscopy, and the time to caecal intubation and withdrawal time are shown in Table 3. The polyp detection rate in the PEG-ELS combined with lubiprostone group was 58.8%, and that in the PEG-ELS alone group was 64.7%. The adenoma detection rate in the PEG-ELS combined with lubiprostone group was 41.2%, and that in the PEG-ELS alone group was 35.3%. There was no clinically significant difference in PDR or ADR between the 2 groups (*p* = 0.62 and *p* = 0.62, respectively).

**Table 3:** Quality indicators for colonoscopy.

	PEG-ELS + Lubiprostone	PEG-ELS alone	*p* value
Polyp detection rate, n (%)	20 (58.8)	22 (64.7)	0.62
Adenoma detection rate, n (%)	14 (41.2)	12 (35.3)	0.62
Caecal intubation time, median (sec) (IQR1, IQR3)	360 (296, 480)	366.5 (267, 504)	0.86
Withdrawal time, median (sec) (IQR1, IQR3)	763.5 (493, 1143)	721.5 (523, 1020)	0.97
Complete colonoscopy, n (%)	34 (100)	34 (100)	–

Adverse events are shown in Table 4. There were no serious adverse events in either group in this study. The common side effects of lubiprostone are nausea and vomiting. In this study, there was no difference in severe symptoms of nausea and vomiting between the PEG-ELS combined with lubiprostone group and the PEG-ELS alone group (severe nausea = 5 (14.7%) vs. 3 (8.8%), *p* value = 0.71; severe vomiting = 1 (2.9%) vs. 1 (2.9%), *p* value = 1.00).

**Table 4:** Adverse events.

	PEG-ELS + Lubiprostone	PEG-ELS alone	*P* value
Anaesthetic complications			
Hypotension, n (%)	2 (5.9)	4 (11.8)	0.34
Symptoms			
Nausea, n (%)	5 (14.7)	3 (8.8)	0.71
Vomiting, n (%)	1 (2.9)	1 (2.9)	1
Bloating, n (%)	3 (8.8)	3 (8.8)	1
Abdominal pain, n (%)	1 (2.9)	1 (2.9)	1
Dizziness, n (%)	3 (8.8)	0	0.24
Chest pain, n (%)	1 (2.9)	1 (2.9)	1
Sleep disturbance, n (%)	0	2 (5.9)	0.49
Metabolic complications, n (%)			
Acute kidney injury	0	0	1
Hyponatremia	1 (2.9)	1 (2.9)	1
Dyskalemia	3 (8.8)	2 (5.9)	1

Patient satisfaction with the bowel preparation protocol is shown in Table 5. There was no difference in acceptance, compliance, or satisfaction between the 2 groups. One patient from the PEG-ELS + lubiprostone group complained of symptoms after taking lubiprostone and scored satisfaction at 1/10.

**Table 5:** Satisfaction.

	PEG-ELS + Lubiprostone	PEG-ELS alone	*P* value
Acceptance	33 (97.1)	32 (94.1)	1.00
Compliance	33 (97.1)	34 (100)	1.00
Satisfaction	31 (91.2)	33 (97.1)	0.61

## Discussion

Colonoscopy is currently considered a standard and the most commonly used method for the screening, diagnosis, and treatment of disease. Inadequate bowel preparation directly affects the adenoma detection rate and adenoma missed rate^10–12^ and reflects interval colorectal cancer^13^. This study is the first to evaluate the effect of lubiprostone in combination with standard PEG-ELS on bowel preparation in patients with chronic constipation, for whom there is currently no recommended method of bowel preparation.

The success of bowel preparation in this study was very low (50% in the study group and 52.9% in the control group). Since the effect was not clearly different, it may be that the duration of the drug may have been too short, resulting in poor efficacy of lubiprostone. This is consistent with a study by Johanson et al.^14^, which found that the drug stimulates bowel movement even if used within 24–48 h, with results from 36.9% to 56.7% in 24 h and 60.7% to 80% in 48 h. Although the results are significantly different, the differences are still small compared with 7 days of use, which showed the maximum effect (spontaneous bowel movements 5.69 vs. 3.46 in placebo; *p* value 0.0001).

Overall, the results show that the quality of bowel preparation in both groups was poor. This was different from the results reported by the latest study from Srinagarind Hospital that evaluated the use of triple-dose PEG-ELS compared with standard split-dose PEG-ELS for bowel preparation in constipation patients and found that triple-dose PEG-ELS achieved successful bowel cleansing compared with split-dose PEG-ELS (91.4% vs. 73.0%, *p* value = 0.042)^5^. In addition, the study by Stengel JZ and Jones DP (2008) comparing single-dose lubiprostone plus split-dose 4 L PEG showed significantly good quality of bowel preparation compared with split-dose 4 L PEG in patients who needed colonoscopy (86% vs. 56%, *p* value 0.001)^15^. The main factor contributing to this study’s poor bowel preparation results was the lower dose of PEG in both groups because PEG was a more effective bowel preparation method compared to the other drugs. Therefore, PEG reduction could affect the quality of bowel preparation. This is supported by the study of Banerjee et al.^16^, in which reduced doses of PEG (1.5 L PEG with lubiprostone, 1 L PEG with lubiprostone, and 2 L PEG with lubiprostone) showed no difference in the quality of bowel preparation (mean Boston bowel preparation scale scores were 7.30 ± 0.25, 7.25 ± 0.26, and 7.44 ± 0.14, respectively). However, the use of split-dose 2 L PEG is still a popular routine practice before colonoscopy because there is evidence for the safety of dehydration and electrolyte imbalance in patients with various chronic diseases and good compliance with more than 4 L split-dose PEG^3^. There may also be contributing factors that may lead to poor bowel preparation quality, including discontinuing the current laxative drug before colonoscopy, inadequate efficacy of instruction to patients about diet, or severity of constipation compared with other studies. However, because the adenoma detection rate was higher than that in recent studies^5^, the adenoma detection rate must be influenced by more than bowel cleaning factors.

In the past 5 years, there have been many studies on bowel preparation, and it has been found that for patients with chronic constipation, bowel preparation is difficult^17^. Even split-dose PEG results in poor bowel preparation quality. Currently, the following drugs for use in bowel preparation are being studied:

1. Bisacodyl plus split-dose 2 L PEG plus simethicone compared with split-dose 4 L PEG showed no significant differences in bowel preparation. However, the combination performed better than the standard regimen in terms of colonic mucosa visualization, patient acceptance and compliance.^3^
2. Lactulose 30 ml plus split-dose 4 L PEG has been shown to be significantly superior to the conventional method for colonoscopy bowel preparation in patients with constipation. However, the Ottawa bowel preparation score improved only to 8.40 ± 0.84^18^.

In the present study, we found no clinically significant difference in efficacy between the 2 regimens, which is different from recent studies that showed that lubiprostone plus PEG-ELS in patients without constipation benefited from improved bowel cleansing^15,16,19^. Studies on the efficacy of lubiprostone in chronic idiopathic constipation have shown that it can improve spontaneous bowel movements in one week and increased in later weeks^14,20^. Therefore, constipation must be one of the major factors that impacts bowel cleansing in combination with lubiprostone, and further studies with an extended duration of lubiprostone use in bowel preparation before colonoscopy can prove this hypothesis.

To our knowledge, our study is the first to evaluate the combination of lubiprostone with split-dose PEG-ELS in constipation patients. However, a limitation is that this study was performed in single and tertiary care settings that may not represent all constipation patients.

## Conclusion

We found no additional benefit for successful bowel preparation from the combination of lubiprostone and PEG-ELS in chronic constipation patients undergoing colonoscopy.

## Data Availability

The datasets used and/or analysed during the current study are available from the corresponding author upon reasonable request.
